# Imaging of Lipid Peroxidation-Associated Chemiluminescence in Plants: Spectral Features, Regulation and Origin of the Signal in Leaves and Roots

**DOI:** 10.3390/antiox11071333

**Published:** 2022-07-06

**Authors:** Michel Havaux, Brigitte Ksas

**Affiliations:** Aix-Marseille University, CEA, CNRS, UMR7265, Bioscience and Biotechnology Institute of Aix-Marseille, CEA/Cadarache, F-13115 Saint-Paul-lez-Durance, France; brigitte.ksas@cea.fr

**Keywords:** lipid peroxidation, reactive oxygen species, singlet oxygen, spontaneous chemiluminescence, spontaneous photon emission, ultraweak light emission, lipoxygenase, detoxification

## Abstract

Plants, like most living organisms, spontaneously emit photons of visible light. This ultraweak endogenous chemiluminescence is linked to the oxidative metabolism, with lipid peroxidation constituting a major source of photons in plants. We imaged this signal using a very sensitive cooled CCD camera and analysed its spectral characteristics using bandpass interference filters. In vitro oxidation of lipids induced luminescence throughout the visible spectrum (450–850 nm). However, luminescence in the red spectral domain (>640 nm) occurred first, then declined in parallel with the appearance of the emission in the blue-green (<600 nm). This temporal separation suggests that the chemical species emitting in the blue-green are secondary products, possibly deriving from the red light-emitting species. This conversion did not seem to occur in planta because spontaneous chemiluminescence from plant tissues (leaves, roots) occurred only in the red/far-red light domain (>640 nm), peaking at 700–750 nm. The spectrum of plant chemiluminescence was independent of chlorophyll. The in vivo signal was modulated by cellular detoxification mechanisms and by changes in the concentration of singlet oxygen in the tissues, although the singlet oxygen luminescence bands did not appear as major bands in the spectra. Our results indicate that the intensity of endogenous chemiluminescence from plant tissues is determined by the balance between the formation of luminescent species through secondary reactions involving lipid peroxide-derived intermediates, including singlet oxygen, and their elimination by metabolizing processes. The kinetic aspects of plant chemiluminescence must be taken into account when using the signal as an oxidative stress marker.

## 1. Introduction

Plants, animals and microbes glow continuously. This spontaneous chemiluminescence (SCL) is attributed to endogenous production of excited states during oxidative metabolic reactions and is therefore closely related to the oxidation status of the emitting organism [1,2,3,4]. SCL has a very low intensity, typically around 10^−19^ to 10^−16^ W cm^−2^ (corresponding to a few hundred photons s^−1^ cm^−2^), i.e., several orders of magnitude less than bioluminescence which arises from enzymatic reactions in some animals, including fireflies, jellyfishes and fishes [5]. Despite the ultraweak nature of SCL, it is possible to image this signal with an adequate spatial resolution using highly sensitive imaging devices such as charge-coupled device (CCD) cameras. Deep cooling of the CCD sensor using liquid nitrogen or some efficient thermoelectric modules is required for reducing thermal noise and enabling detection of faint light using long acquisition times. Because SCL imaging can be performed non-invasively on intact organisms or organs and does not require any exogenously applied probe, it is expected to provide realistic mapping of oxidative stress, and it has many potential applications.

Lipid peroxidation has been identified as a major source of SCL [1,2,3,4,6]. This is particularly true in plants because their lipids are highly unsaturated [7,8] and are therefore very sensitive to oxidation [9]. In general, lipids constitute primary targets of reactive oxygen species and oxidative stress in plants. Accordingly, plant mutants more sensitive to lipid oxidation, such as Arabidopsis mutants deficient in tocopherol (*vte1)* [2,10] or lacking the chloroplastic lipocalin LNCP [11,12], emit more photons than do wild types. Moreover, SCL intensity is usually well correlated with the extent of lipid peroxidation measured by classical biochemical methods (e.g., [13,14]). However, the by-products of lipid peroxidation that are responsible for SCL in plants have not yet been identified with certainty. A number of candidates have been proposed, such as singlet oxygen and triplet excited carbonyls, which can both emit photons of visible light [15]. The former molecule can be produced in reactions between two lipid peroxy radicals, while the latter compounds can be formed by a number of secondary reactions such as the self-reaction of alkoxy radicals or dioxetane decomposition. However, the causal relationship between SCL emission and the formation of those compounds in lipid peroxidation has not yet been demonstrated unambiguously. 

In this work, SCL was imaged in plant leaves and roots under conditions that induced lipid peroxidation, and the spectral characteristics of the imaged signal were measured from 400 to 850 nm. Leaf or root SCL emission was found to occur only in the red- and far-red wavelength ranges. The SCL spectrum was also observed to be independent of chlorophyll. The intensity of the signal was modulated by the concentration of singlet oxygen and by cellular detoxification capacities in the plant tissues. Based on those results, a model for the origin of plant SCL is proposed.

## 2. Materials and Methods

### 2.1. Plant Material and Treatments

Arabidopsis plants (Arabidopsis thaliana, ecotype Col-0) were grown for 4–5 weeks in a phytotron in the Phytotec platform of the BIAM Institute (CEA/Cadarache) under controlled conditions: temperature, 24/20 °C (day/night); photon flux density, 120 µmol photons m^−2^ s^−1^; photoperiod, 8 h; air humidity, 55%. The following mutant lines were also used: the variegated *thf1* mutant [16], the lipoxygenase2-deficient *lox2-1* mutant [17] and the transgenic luminescent line *pAER:LUC* [18]. Pea seeds (Pisum sativum) were germinated on wet filter paper in Petri dishes for 3 d. Pea, rather than Arabidopsis, was used for root analyses because Arabidopsis roots are too small to be easily injured with a scalpel and for their luminescence to be easily measured with this treatment.

Germinated pea seeds were incubated in water (control), 70% deuterium oxide, 20 mM histidine, 25 mM ascorbate or 6 mM Trolox (6-hydroxy-2,5,7,8-tetramethylchroman-2-carboxylic acid) for 3 h before wounding. Histidine, ascorbate and Trolox are singlet oxygen scavengers [19], whereas deuterium oxide is an enhancer of singlet oxygen lifetime [20]. Roots were injured with a scalpel and leaves were wounded with a pair of tweezers. Droplets of water, 70% deuterium oxide, 20 mM His, 25 mM ascorbate or 6 mM Trolox were deposited on wounded leaf areas. 

Arabidopsis leaf discs (diameter, 14 mm) were exposed for 18 h to high light (1400 µmol photons m^−2^ s^−1^) at 7 °C.

### 2.2. Oxidation of Lipids In Vitro

Sunflower oil was aerated for 24 h by vigorous stirring using a magnetic stirrer and a magnetic stir bar. Sunflower oil (1 mL) was also oxidized by the Fenton reaction by adding 5 mM FeCl_2_ (100 µL) and 30% H_2_O_2_ (60 µL). Linolenic acid (Fluka, 3.8 mg mL^−1^) was enzymatically oxidized with soybean LOX (Fluka, 5 mg mL^−1^) in 100 mM borate buffer pH 9 (KOH), as previously described [6]; 1 mL linolenic acid was mixed with 1.5 mL LOX. An amount of 1 mL of the different mixtures was then placed in 5-mL glass beakers for luminescence imaging. Except for time-dependence analyses of the signal, the measurements were performed 15–20 min after the start of the reactions. 

### 2.3. Luminescence Imaging

The luminescence emissions from plants, leaf discs or liquid solutions were imaged using a highly sensitive, liquid N_2_-cooled charge coupled device (CCD) camera (VersArray 1300 B, Roper Scientific). The camera was mounted with a back-illuminated CCD (CCD3640, e2v Technologies, Essex, UK) with a 1340 × 1300 imaging array showing a quantum efficiency (QE) peaking at 560 nm (QE = 0.93) and higher than 0.75, between 425 and 770 nm. Above 770 nm, the sensitivity of the sensor monotonously declines, reaching a QE of 0.1 at ca. 1000 nm. The sensor operates at a temperature of −110 °C. The sample was imaged on the sensor by a 50-mm focal distance lens with an f-number of 1.2 (F mount Nikkor 50-mm, f:1.2, Nikon) for maximizing light collection. The camera was mounted on a laboratory-built black box and was placed in a dark room to avoid contamination by external photons. In general, acquisition time for the imaging of plant material was 20 min (except for spectral analyses; see below), with on-CCD binning of 2 × 2 or 3 × 3. For the imaging of oxidized lipids in solution, binning was 5 × 5. 

A filter holder was attached to the camera lens, and different types of optical filters were used to analyse the spectral characteristics of the measured signal: a long wave pass filter, Corion LG640 (cut-on wavelength for 50% transmission, 640 nm) and a short wave pass filter, Corion LS600 (415–600 nm). For a more resolved spectral analysis of the signal, bandpass interference filters with a bandwidth of 50 nm (Omega Optical, 425-, 475-, 525-, 575-, 625-, 675-, 725-, 775-, 825-BP50 filters, diameter 4.6 cm) were placed in front of the lens system of the camera. We checked that none of the filters are luminescent. Pixel binning of 5 × 5 and an acquisition time of 5 min were used for the image recordings. The bandpass of the optical filters has very sharp edges (<10 nm from 0 to maximum transmission) and a high % transmission (>90%). Because plant autoluminescence intensity decreases with time, imaging was carried out rapidly after treatment. Wounding of plant leaves was performed in dim light after a period of dark adaptation to avoid activation of chlorophyll luminescence. Under those conditions, background autoluminescence of Arabidopsis leaves was very low and did not interfere significantly with the measurements of wound-associated luminescence. In general, images were recorded a maximum of 30 min after plant treatment.

The same imaging system was used for recording the luciferin/luciferase luminescence from the *pAER:LUC* line. Plants were sprayed with a 5 mM aqueous solution of luciferin containing 0.01% Triton 100. Acquisition time was 3 min, and the on-CCD pixel binning was 2 × 2. All images were analysed using Image J software. 

### 2.4. Hydroxy Fatty Acid Measurements

Lipid peroxidation was measured by quantifying HOTEs (hydroxyoctadecatrienoic acids, C18:3-OH) and HODEs (hydroxyoctadecadienoic acid, C18:2-OH), oxidation products of linolenic acid (C18:3) and linoleic acid (C18:2), respectively [21,22]. Lipids were extracted from leaf samples (*ca*. 300 mg fresh weight) in methanol/chloroform, and HOTEs and HODEs were quantified by HPLC-UV by distinguishing LOX-dependent HOTEs from ROS-induced HOTEs, as detailed elsewhere [21,22].

### 2.5. SOSG-EP Fluorescence Imaging and Spectroscopy

Attached leaves were infiltrated with a singlet oxygen-specific fluorescent probe SOSG (30 µg mL^−1^), as previously described [23]. Germinated pea seeds were incubated in the SOSG solution for 4 h. Leaves and roots, pre-treated with SOSG, were wounded with a pair of tweezers (leaves) or with a scalpel (roots). SOSG endoperoxide (SOSG-EP) fluorescence, resulting from the interaction of SOSG with singlet oxygen, was imaged with a Zeiss Axiozoom V16 microscope. Excitation light wavelengths were in the range of 450–490 nm, and fluorescence emission was measured at 500–550 nm. 

SOSG-EP fluorescence spectra were measured with a Perkin-Elmer LS50 spectrofluorometer equipped with fibreoptics. Fluorescence was excited with a 470 nm light. 

The SOSG probe has known limitations. Its main shortcoming is its sensitivity to light and its ability to act as a photosensitizer capable of generating singlet oxygen in the light. However, these effects can be considered as negligible in this study because SOSG was used to measure the singlet oxygen produced by wounding in the dark or under very low light. The penetration of the probe inside the plant cells is also a crucial factor. Consequently, the SOSG method should not be considered as a quantitative method; it is used here only to detect the formation of singlet oxygen in wounded tissues.

### 2.6. Hydroxy-Plastochromanol Measurements

Leaves were injured with tweezers and frozen in liquid nitrogen. The samples (~50 mg) were ground in ethyl acetate. After centrifugation, the supernatant was filtered and evaporated on ice under a stream of N_2_. The residue was recovered in methanol/hexane (17/1) and analysed by HPLC, as described elsewhere [10]. The column was a Phenomenex Kinetex 2.6 μm, 100 × 4.6 mm, 100 Å. The HPLC analysis was performed in the isocratic mode with methanol/hexane (17/1) as a solvent system and with a flow rate of 0.8 mL min^−1^. Hydroxy-plastochromanol-8 was detected by its fluorescence at 330 nm with an excitation at 290 nm. The standard was a kind gift from Dr. J. Kruk (Krakow, Poland). 

## 3. Results

### 3.1. Spectral Characteristics of Plant Leaf SCL

**Figure 1:**
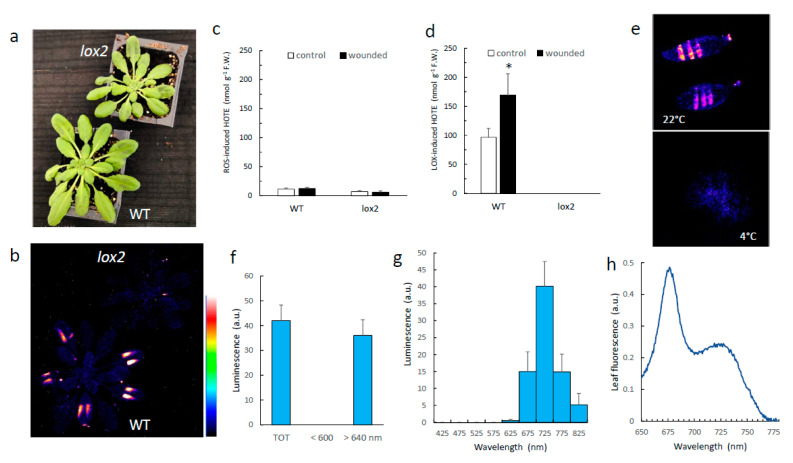
SCL emission induced by leaf wounding in WT Arabidopsis plants and in the *lox2* mutant. (**a**) Picture of the plants after leaf wounding. (**b**) SCL images of the corresponding plants. The colour palette indicates the signal intensity from low (dark blue) to high (white) values. (**c**,**d**) ROS-mediated and LOX-mediated HOTE levels in control leaves and in wounded leaves. *, significantly different from WT at *p* < 0.01 (Student’s *t*-test). (**e**) Wounding-induced SCL in WT Arabidopsis leaves at 22 °C and at 4 °C. (**f**) Wounding-induced SCL intensity in two wavelength ranges, >640 nm and <600 nm. (**g**) Spectrum of wounding-induced luminescence in WT leaves. (**h**) Chlorophyll fluorescence emission of a WT Arabidopsis leaf at room temperature. TOT = total signal intensity.

Wounding Arabidopsis leaves (e.g., using a pair of tweezers as in Figure 1a) is known to induce lipoxygenase activity [24,25], and this causes the oxidation of fatty acids, as shown in Figure 1d, by the accumulation of HOTEs, the primary oxidation products of linolenic acid. Accordingly, the effect of wounding in WT leaves was observed on the LOX-mediated HOTE levels (Figure 1d), while HOTEs resulting from ROS attack on linolenic acid did not change (Figure 1c). This phenomenon is associated with the induction of a luminescence signal at the place of injury (Figure 1b), as previously reported [6,26,27]. The causal relationship between LOX activity and wound-induced SCL emission is confirmed by the complete absence of light emission in the *lox2* mutant (Figure 1b). Wounding-induced SCL was temperature-dependent; almost no luminescence was measured at 4 °C (Figure 1e), in line with the enzymatic origin of the signal. 

Using long and short wave pass filters, we found that wound-induced SCL emission occurs in the red spectral domain above 640 nm. No signal was measured at wavelengths below 600 nm (Figure 1f). More resolved spectra were obtained using bandpass interference filters with a 50-nm bandwidth, showing that leaf SCL peaks in the far-red spectral domain between 700 and 750 nm (Figure 1g). This luminescence spectrum shows some similarities with the spectrum of chlorophyll fluorescence, which also occurs in the red and far-red spectral domains (see, e.g., [28]). However, in vivo chlorophyll fluorescence from Arabidopsis leaves (Figure 1h) did not closely match the SCL spectrum of the corresponding leaf (Figure 1g): chlorophyll fluorescence was maximal at around 680 nm, thus exhibiting a shift towards lower wavelengths compared to the wound-induced SCL peak. 

**Figure 2:**
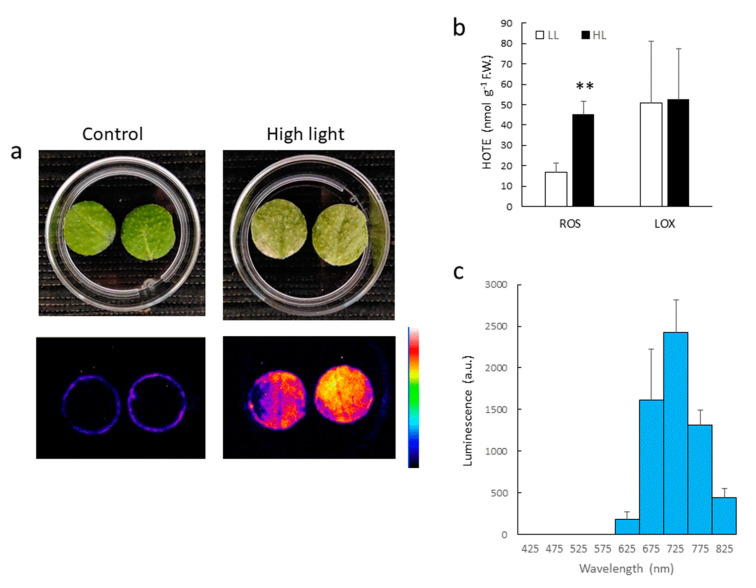
SCL emission induced by a high light treatment of WT Arabidopsis leaf discs. (**a**) Picture and SCL image of control and high light-treated leaf discs. (**b**) ROS- and LOX-mediated HOTE accumulation. **, significantly different from LL at *p* < 0.005 (Student’s *t*-test). (**c**) SCL spectrum of high light-treated leaf discs. Leaf discs were exposed to white light of PFD 1400 µmol m^−2^ s^−1^.

Leaf SCL is not specific to wounding, but it can be induced by any pro-oxidative conditions [2]. For instance, photooxidative stress, induced by strong light combined with low temperature, induced luminescence emission in Arabidopsis leaf discs (Figure 2a) in parallel with lipid peroxidation and HOTE accumulation (Figure 2b). Contrary to wounding, HOTEs originated from ROS attack on lipids rather than by LOX activity. Spectral analysis of the SCL emissions showed that high light (Figure 2c) and wounding (Figure 1g) resulted in quite similar spectra peaking in the region of 700–750 nm. Again, no luminescence was detected below 600 nm. 

### 3.2. Luminescence of Lipids Oxidized In Vitro

**Figure 3:**
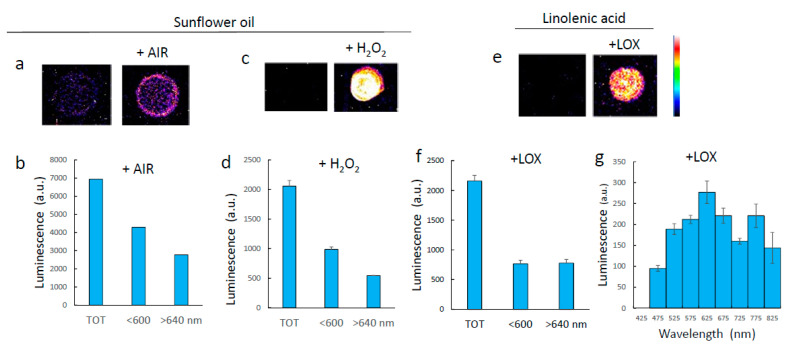
Luminescence imaging of lipids oxidized in vitro. (**a**) Luminescence image of sunflower oil oxidized by aeration under stirring. (**b**) Luminescence intensity of aerated sunflower oil at wavelengths <600 nm and >640 nm. (**c**) Luminescence image of sunflower oil oxidized by hydrogen peroxide in the presence of iron. (**d**) Luminescence intensity of hydrogen peroxide-treated sunflower oil at wavelengths <600 nm and >640 nm. (**e**) Luminescence image of linolenic acid oxidized by soybean LOX. (**f**) Luminescence intensity of oxidized linolenic at wavelengths <600 nm and >640 nm. (**g**) Luminescence spectrum of oxidized linolenic acid. TOT = total signal intensity.

In Figure 3, lipids in solution were oxidized by different means. Sunflower oil was aerated under vigorous stirring for 24 h, causing some oxidation, as shown by the appearance of a weak luminescence (Figure 3a). Oxidation was strongly amplified when sunflower oil was treated with hydrogen peroxide in the presence of iron (producing hydroxyl radicals by the Fenton reaction) (Figure 3c). In both cases, luminescence occurred in both the red (>640 nm) and the blue-green (<600 nm) spectral domains (Figure 3b,d). Linolenic acid was also oxidized in vitro with soybean LOX (Figure 3e). Similarly to sunflower oil, the luminescence of enzymatically oxidized linolenic acid covered a large part of the visible spectrum, both <600 nm and >640 nm. A more detailed spectral analysis showed that luminescence occurred throughout the visible domain from 450 to 850 nm, with two small maxima at 600–650 nm and at 750–780 nm. 

### 3.3. Non-Chlorophyllous Material

**Figure 4:**
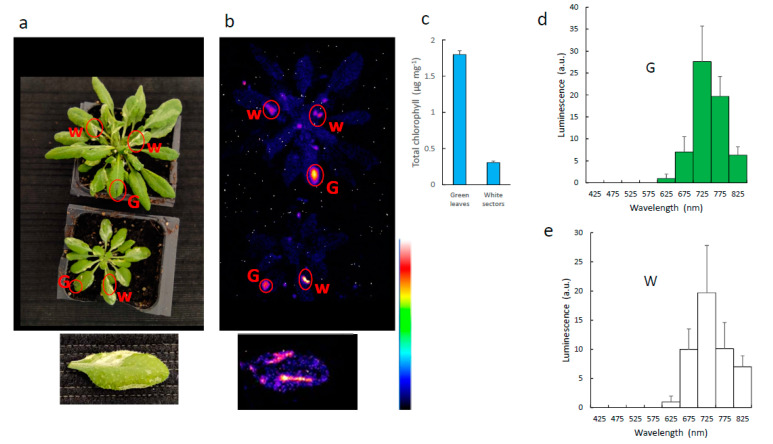
Wounding-induced SCL emission from leaves of the variegated *thf1* Arabidopsis mutant. (**a**) Picture of *thf1* plants. Green (G) and white (W) leaf sectors were wounded with tweezers. (**b**) SCL image of the plants. At the bottom of panels (**a**,**b**), there is a close-up shot of a *thf1* leaf. One incision has been made with a scalpel on the green and the white parts of the leaf. (**c**) Chlorophyll is drastically decreased in the white sectors of *thf1* leaves. (**d**,**e**) SCL spectrum of wounded green and white leaf sectors.

The luminescence characteristics of oxidized lipids (Figure 3) are different from those of leaf SCL (Figure 1 and Figure 2), although lipid oxidation is the main source of photon emission in plants [6]. A previous hypothesis was that, in green plants, chlorophyll could be the final in vivo emitter [29,30] by the transfer of excitation from excited species, such as triplet carbonyls, to chlorophyll [31,32]. To check this hypothesis, we examined non-chlorophyllous plant materials. In Figure 4, we measured the effects of wounding on leaf SCL in the variegated Arabidopsis mutant *thf1*. Both green and white leaf sectors showed luminescence upon wounding (Figure 4b), and the emission spectra were very similar (Figure 4d,e), with maximal emissions around 725 nm. SCL intensity levels from white and green sectors cannot be directly compared because their relative lipid composition and LOX activity are not known. However, they were not dramatically different (Figure 1b). 

**Figure 5:**
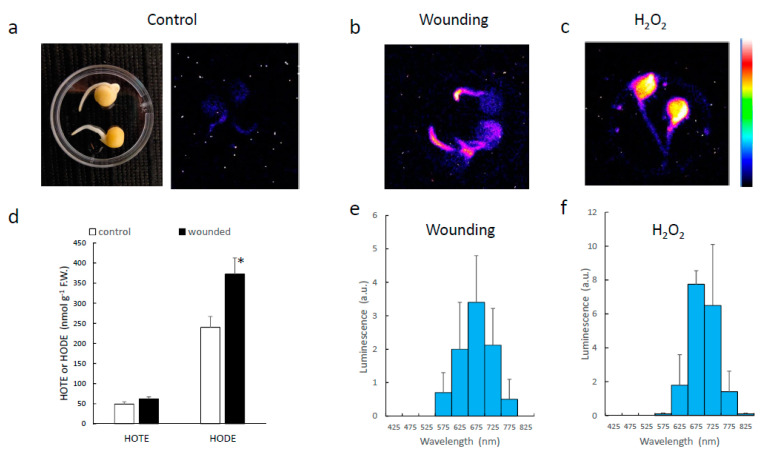
SCL in pea roots. (**a**) SCL emission from control pea seeds. Seeds were left to germinate on wet filter paper for 3 d. (**b**) SCL of pea seeds with wounded roots. (**c**) SCL of seeds exposed for 4 h to hydrogen peroxide. (**d**) HOTE and HODE levels in pea roots before and after wounding. *, significantly different from control at *p* < 0.01 (Student’s *t*-test). (**e**) SCL spectrum of wounded pea roots. (**f**) SCL spectrum of pea roots exposed to hydrogen peroxide.

The boundary between green and white areas in *thf1* leaves is blurred (bottom of Figure 1a), so that it is difficult to injure the white zones exclusively, and low levels of chlorophyll are still present in the wounded areas (Figure 4c). Therefore, we also imaged the SCL emissions from pea roots (Figure 5), which are completely devoid of chlorophyll. Luminescence from intact roots was hardly visible with our system (Figure 5a). Wounding roots with a scalpel brought about a noticeable enhancement of luminescence (Figure 5b), in accordance with early investigations on injured soybean roots [33]. This phenomenon was accompanied by HOTE and HODE accumulation (Figure 5d). The high levels of HODE (oxidation product of linoleic acid) relative to HOTE is indicative of the high levels of linoleic acid in roots compared to leaves [8]. Incubation of pea seeds in hydrogen peroxide and FeCl_2_ also triggered SCL (Figure 5c). In both conditions, luminescence took place exclusively in the red at 650–750 nm (Figure 5e,f). No photon emission was detected <600 nm. We can thus conclude from the results of Figure 4 and Figure 5 that the spectrum of in vivo SCL does not depend on chlorophyll. 

### 3.4. Singlet Oxygen

**Figure 6:**
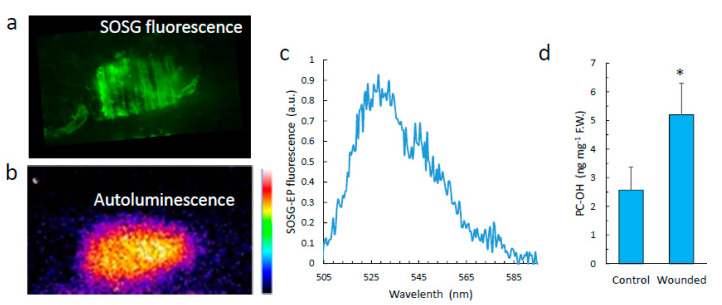
Singlet oxygen in wounded Arabidopsis leaves. (**a**) SOSG-EP fluorescence in a wounded Arabidopsis leaf. (**b**) SCL image of the wound shown in panel (**a**). (**c**) Spectrum of the fluorescence emitted by the wounded leaves pre-infiltrated with SOSG. (**d**) Hydroxy-plastochromanol (PC-OH) levels in control and wounded leaves. *, significantly different from control at *p* < 0.01 (Student’s *t*-test).

**Figure 7:**
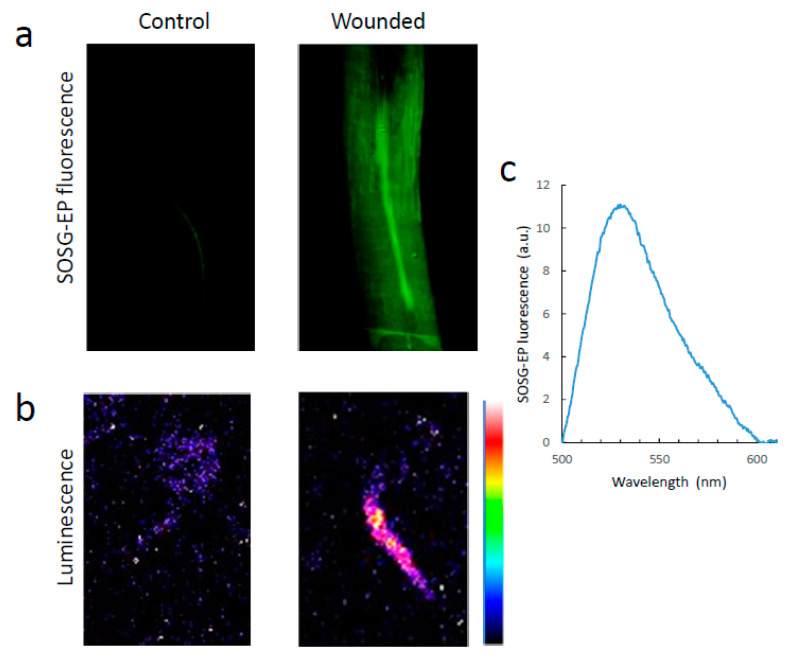
Singlet oxygen in wounded pea roots. (**a**) SOSG-EP fluorescence in a wounded pea root. (**b**) SCL image of the wound shown in panel (**a**). (**c**) Spectrum of the fluorescence emitted by wounded roots pre-treated with SOSG.

It has been shown that leaf wounds or high light-treated leaves generate singlet oxygen [23,34]. Singlet oxygen luminesces in the near infra-red at 1270 nm, but its dimol emission has bands in the red, at 634 nm and 703 nm [15]. In Figure 6, we imaged singlet oxygen in leaves infiltrated with the singlet oxygen-sensitive fluorescence probe Singlet Oxygen Sensor Green (SOSG). Upon reaction of SOSG with singlet oxygen, an endoperoxide (SOSG-EP) is formed, generating a green fluorescence in the range of 525–536 nm when excited at around 500 nm [35]. The leaf sector wounded with tweezers clearly exhibited the fluorescence of SOSG-EP, indicating singlet oxygen production in this zone. The fluorescence spectrum shown in Figure 6c has a peak at ca. 530 nm, typical of SOSG-EP fluorescence. Figure 6a shows the SCL image of the corresponding area of the leaf. A similar experiment was performed on pea roots (Figure 7). The injuries induced on the roots with a scalpel emitted SOSG-EP fluorescence (Figure 7a), peaking at 530 nm (Figure 7c). 

Hydroxy-plastochromanol, a singlet oxygen marker in Arabidopsis [36], was measured in control and wounded leaves (Figure 6d). Wounding significantly increased the concentration of hydroxy-plastochromanol, in agreement with the formation of singlet oxygen (Figure 6b,c).

**Figure 8:**
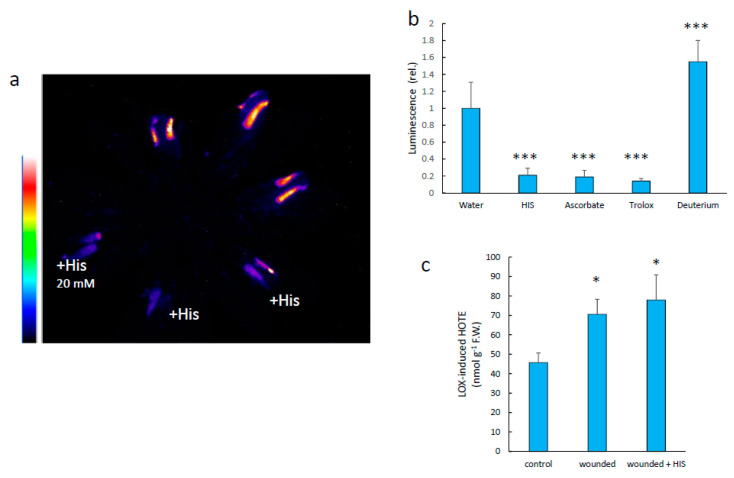
Effect of singlet oxygen quenchers on the intensity of Arabidopsis leaf SCL. (**a**) SCL image of an Arabidopsis plant with wounded leaves and the effect of several singlet oxygen quenchers (His, Trolox, ascorbate). (**b**) quantification of leaf SCL under the conditions shown in panel (**a**). (**c**) HOTE levels in control and wounded leaves treated or not with His. ***, significantly different from water at *p* < 0.001; *, significantly different from control at *p* < 0.01 (Student’s *t*-test).

**Figure 9:**
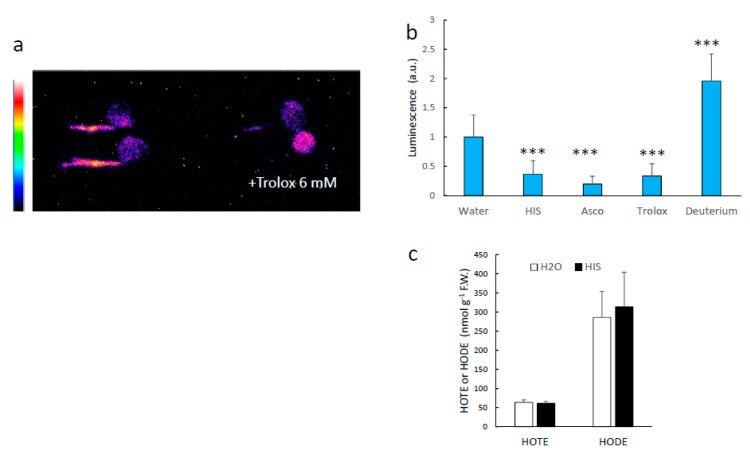
Effect of singlet oxygen quenchers on the intensity of pea root SCL. (**a**) SCL image of wounded pea roots and the effect of several singlet oxygen quenchers (His, Trolox, ascorbate). ***, significantly different from water at *p* < 0.001 (Student’s *t*-test). (**b**) quantification of root SCL under the conditions shown in panel a. (**c**) HOTE and HODE levels in wounded roots treated or not with His.

We checked whether modulating singlet oxygen concentration in plant tissues affects the intensity of the SCL signal (Figure 8). Different singlet oxygen quenchers (histidine, ascorbate, Trolox) were applied on the wounds, causing a marked decrease in luminescence (Figure 8a,b). This effect occurred without significant changes in the HOTE levels (Figure 8c), indicating that the quenchers did not interfere directly with the lipid oxidation step itself. On the other hand, treating the wounds with deuterium oxide, a compound that lengthens the lifetime of singlet oxygen [20], slightly enhanced the SCL signal (Figure 8b). The same response to singlet oxygen quenchers or deuterium was observed in injured pea roots (Figure 9). It thus appears that singlet oxygen is involved in the SCL signal, either directly or indirectly.

### 3.5. Detoxification of Lipid Peroxidation Products

**Figure 10:**
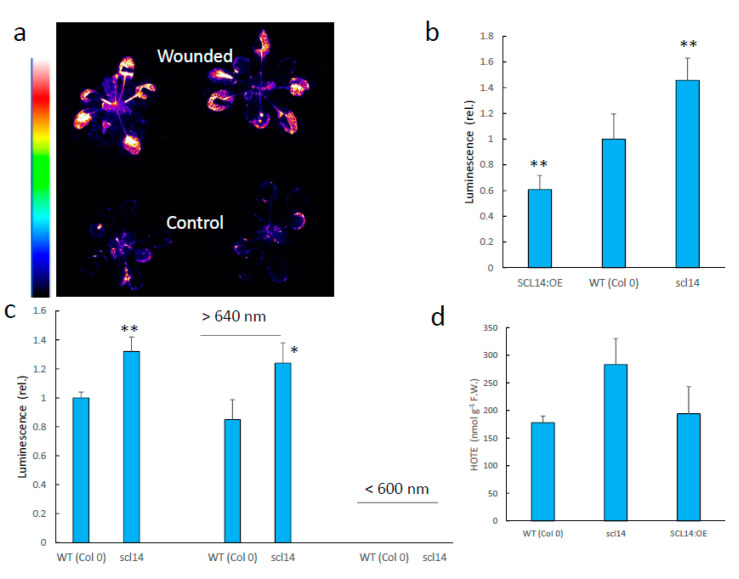
Leaf wounding and cellular detoxification capacities. (**a**) Effects of leaf wounding on the luciferin/luciferase luminescence of Arabidopsis *pAER:LUC* plants. (**b**) Intensity of wounding-induced leaf SCL in WT Arabidopsis and in the *scl14* mutant and the *SCL14:OE*. (**c**) wounding-induced SCL in two spectral domains (<600 nm and >640 nm). (**d**) HOTE levels. * and **, significantly different from WT at *p* < 0.01 and 0.005, respectively (Student’s *t*-test).

Lipid hydroperoxides are labile and spontaneously decompose into various derivatives, including reactive carbonyl species (RCS) [37,38,39]. To cope with the toxicity of some of those derivatives, plant tissues contain various detoxifying enzymes, such as those that convert RCS into less toxic compounds such as alcohols or carboxylic acids [38,39]. In particular, alkenal reductase (AER) is one such enzyme which targets several lipid oxidation metabolites with a particular affinity for hydroxynonenal (HNE) [40,41]. Figure 10a shows the effect of wounding on AER expression using the *pAER:Luc* line that expresses the LUC gene, coding for the luciferase enzyme, under the control of the AER promoter [18]. Spraying *pAER:Luc* plants with luciferin induces a luminescence signal if luciferase is present, i.e., under conditions promoting AER expression. Wounding brought about a marked increase in the luciferase/luciferin luminescence, indicating upregulation of AER. This result is consistent with previous transcriptomic analyses of the Arabidopsis response to wounding, which have shown induction of various detoxification-related genes such as aldehyde reductase, glutathione reductases, cytochromes P450, etc. [42]. 

AER and a number of detoxifying enzymes belong to the so-called xenobiotic response, which is governed by class-II TGA transcription factors interacting with the SCL14 (SCARECROW-LIKE 14) regulator [43]. We used the Arabidopsis *scl14* mutant and the SCL14:OE overexpressor to analyse the impact of cellular detoxification mechanisms on luminescence in wounded leaves. In the *scl14* mutant, the xenobiotic detoxification pathway is down-regulated [43,44]. On the other hand, the pathway is constitutively induced in the SCL14 over-expressor, and responds more strongly to inducers [43,44]. Interestingly, the intensity of wounding-induced SCL was inversely correlated with the SCL14 levels and the activation of the detoxification pathway (Figure 10b). This correlation did not apply to the HOTE levels, which did not significantly differ between WT and *scl14* after wounding (Figure 10d), confirming that SCL14 acts downstream of the initial lipid oxidation events. As shown above for WT leaves, the increased SCL in wounded *scl14* leaves took place at wavelengths > 640 nm. Therefore, the SCL14-controlled detoxification process targets species that emit in the red wavelength region. 

**Figure 11:**
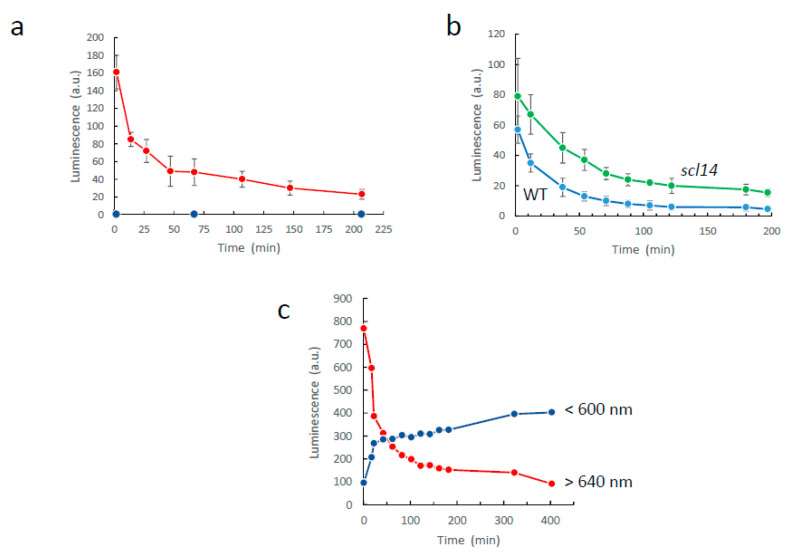
Stability of lipid peroxidation-related SCL. (**a**) Time course of the change in endogenous chemiluminescence in wounded Arabidopsis leaves: in red, total emission; in blue, emission at wavelengths <600 nm. (**b**) Comparison of the time dependence of SCL from wounded WT and *scl14* leaves. (**c**) Time course of the changes in luminescence emission from oxidized lipids in the blue-green (<600 nm) and red/far-red (>640 nm) light domains. Linolenic acid was oxidized by soybean LOX as in Figure 3e–g. The blue-green and (far-)red signals were measured simultaneously by placing a LG640 filter or a LS600 filter above the lipid solutions in the black box of the CCD camera installation.

Leaf SCL decreases over time [6,34], presumably as a result of inhibition of the lipid oxidation process, consumption of the formed lipid peroxides and/or metabolization of the emitting chemical species. The decline in the intensity of wound-induced SCL in Arabidopsis leaves is shown in Figure 11a. Luminescence substantially decreased during the first 10 min after wounding, then a continual, slower decrease was observed. These variations are exclusively due to the red luminescence because no photon emission was measured at wavelengths < 600 nm, even immediately after wounding (<2 min). The time dependence of SCL from wounded WT leaves was compared with that of wounded *scl14* leaves (Figure 11b). SCL showed that the enhanced luminescence intensity of *scl14* was due, at least partially, to a slower decline of the signal. 

We also monitored the time course of decline of the luminescence signal emitted by oxidized lipid solutions (Figure 11c). After oxidation by LOX, linolenic acid generates chemical species that emit photons, both in the red or in the blue-green (Figure 3). The two signals were measured simultaneously, and they exhibited very different kinetics of appearance and decline. The red luminescence appeared first and then waned over time. The blue-green luminescence appeared in a second phase, in parallel with the decline of the red signal. The inverse temporal relationship between the intensity of the red and the blue-green signals gives rise to the possibility that the blue-green emitters are secondary products derived from the red-emitting products. 

## 4. Discussion

SCL emission from plant leaves and roots occurs in the red/far-red light domain at around 700–750 nm, independently of the presence of chlorophyll. Thus, our results do not support the idea that chlorophyll is the final emitter of this luminescence in plants. Moreover, the SCL signal peaks at wavelengths higher than the maximum of in vivo chlorophyll fluorescence (680 nm). We have previously shown that lipid peroxidation is a major source of SCL in plants [6], and this is confirmed here: the signal was correlated with the induction of ROS- or LOX-mediated accumulation of hydroperoxy fatty acids, and blocking lipid peroxidation in the Arabidopsis *lox2* mutant cancelled the signal emission. Therefore, the observation that in vitro oxidized lipids emit photons throughout the visible spectrum, not only in the red/far-red region, may appear as contradictory. Similar spectra, peaking at approximately 600 nm, were previously obtained with linolenic acid oxidized by ultraviolet-A irradiation [45]. However, a kinetic analysis of the induction and subsequent decline of in vitro luminescence emission from oxidized lipids revealed that the signals emitted at wavelengths >640 nm and <600 nm do not appear simultaneously. The red signal is first emitted, then declines in parallel with the appearance of the blue-green signal. This inverse relationship suggests that the latter signal could come from secondary products derived from the red-emitting compounds. This conversion does not appear to take place in planta because photon emission below 600 nm was detected neither at a time very close to induction, e.g., almost immediately after leaf wounding, nor after a long period in darkness. 

The hydroperoxides formed during lipid peroxidation are known to decompose in a variety of secondary products [37,39]. Among these by-products, the luminescent ones have not yet been characterized in detail. Triplet excited carbonyls and singlet oxygen have repeatedly been presented as possible candidates [46,47]. Triplet carbonyls emit photons in the blue while luminescence of singlet oxygen occurs in the near infra-red (1270 nm), with minor emission bands in the red (633 nm, 703 nm) for the dimol emission [48,49]. The rather flat spectrum of oxidized linolenic acid from 450 nm to 850 nm indicates that the phenomenon underlying the emission is complex and is unlikely to be ascribable exclusively in terms of triplet carbonyl and/or singlet oxygen formation. None of the two phosphorescence bands of bimolecular singlet oxygen in the visible domain were observed in the SCL spectra of leaves or roots as major luminescence signals. Luminescence in the 600–700 nm range represented only ~20% of the total emission. We nevertheless observed an effect of singlet oxygen on the signal intensity. Decreasing singlet oxygen concentration in plant tissues using chemical quenchers markedly lowered leaf and root SCL, whereas increasing singlet oxygen levels enhanced the signal. Moreover, the conditions that triggered leaf/root SCL were associated with singlet oxygen formation. If we consider, based on the spectra, that in vivo luminescence does not emanate directly from singlet oxygen itself, the modulation of the signal intensity by singlet oxygen suggests its involvement in reactions that lead to the formation of the red-emitting species. 

Singlet oxygen can be formed during lipid peroxidation by the Russell mechanism, in which two peroxyl radicals react to generate a tetraoxide intermediate whose decomposition generates singlet oxygen [15]. Under osmotic stress, Arabidopsis roots have been shown to produce singlet oxygen, which is dependent on LOX activity and the accumulation of fatty acid hydroperoxides, thus supporting the participation of a Russell mechanism in light-independent singlet oxygen generation in plant tissues [50]. However, other pathways for the formation of singlet oxygen from hydroperoxides have been reported [47]. Singlet oxygen is a highly electrophilic compound that readily reacts with carbon–carbon double bonds in biological molecules [51], so that various secondary products can be produced by singlet oxygen-mediated oxidation during the lipid peroxidation process. Actually, reaction between singlet oxygen produced through lipid peroxidation and biomolecules in wounded leaves is confirmed by the increase in hydroxy-plastochromanol levels. This compound is a specific marker of singlet oxygen, resulting from the interaction of plastochromanol with singlet oxygen [36,52]. Hydroxy-plastochromanol is particularly useful as a marker of low production levels of singlet oxygen [36]. We hypothesize that some of the products generated by singlet oxygen in wounded leaves are unstable, spontaneously decomposing or decaying radiatively, resulting in the emission of photons measured at 640 nm and above. The fact that plant SCL was not detected at wavelengths below 600 nm does not support a direct involvement of triplet excited carbonyls, which emit at wavelengths around 460–470 nm [15]. SCL emission in the red/far-red wavelength range above 650 nm, with a maximum at 720–750 nm and virtually no emission below 600 nm, was also reported in dark-adapted spinach leaves [29] and in cowpea leaves in response to viral infection [53]. Using broadband-coloured filters, Flor-Henry et al. [27] also reported that the bulk of the photons emitted by wounded Arabidopsis leaves have a wavelength higher than 700 nm. However, the spectral characteristics of SCL are different in animal and human tissues. For instance, spontaneous photon emission from human skin is maximal at around 625 nm [54] and is shifted towards lower wavelengths after UVA irradiation [45]. The SCL emitters are probably not the same in different types of biological tissues and may be dependent on the oxidative conditions that induce SCL. 

In this study, we found another factor that impacts on SCL. Modulating the xenobiotic response pathway has a noticeable effect on the luminescence intensity. Inhibition of the pathway by mutational suppression of the SCL14 regulator enhanced the signal, whereas boosting the pathway by SCL14 overexpression decreased its intensity. Gibberellic-acid insensitive (GAI), Repressor of GAI (RGA) and SCARECROW (SCR) transcription factor family proteins (GRAS), including SCL14, are part of the Rossmann fold superfamily of methyltransferases [55]. However, plant representatives of this family lack the residues necessary for methyltransferase activity [56], implying other activities. SCL14 has been shown to regulate a general broad-spectrum detoxification network by interacting with class-II TGA transcription factors [43]. The SCL14-TGA system governs the expression of various detoxifying enzymes, such as reductases, dehydrogenases and glutathione transferases, which play an important role in the metabolization of exogenous or endogenous toxic compounds [43,44,57]. Lipid oxidation-derived metabolites are among the targets of this pathway [18]. Therefore, we can propose that some of the lipid peroxidation products that emit photons or whose decomposition generates light-emitting species are targeted by the SCL14-dependent detoxification pathway. Because the SCL spectra were similar in WT and *scl14* plants, lack of blue-green luminescence in Arabidopsis leaves is not attributable to a selective detoxification of chemical species emitting photons in this wavelength range; otherwise, we would have observed the appearance of light emission at wavelengths <600 nm when the detoxifying pathway was inhibited in *scl14*. 

## 5. Conclusions

**Figure 12:**
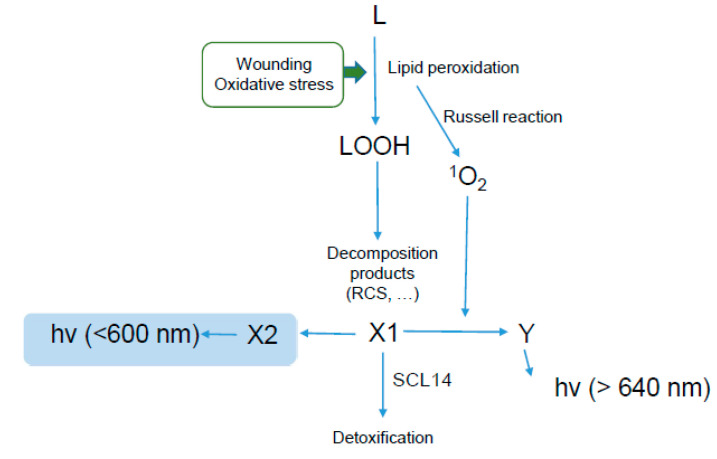
Working model for the origin of SCL emission by plant tissues. Hydroperoxides (LOOH) produced in lipid (L) peroxidation decompose in secondary products symbolized by X1. Reaction between X1 or derived metabolites and singlet oxygen generate species (Y) that luminesce >640 nm. Those luminescent compounds have a rather long lifetime, regulated by the SCL14-dependent detoxification pathway. X1 can lead to X2 (without excluding the possibly that it occurs through Y). X2 or derived metabolites emit photons <600 nm; they accumulate in vitro (highlighted in blue), not in planta.

The scheme of Figure 12 summarizes the main results of the present work and proposes a working model for the origin of plant SCL. Taken together, our results indicate that plant SCL results from secondary reactions involving lipid peroxide-derived intermediates including singlet oxygen. The amplitude of plant SCL provides information on the intensity of lipid peroxidation, but it is also dependent on the efficiency with which the products of lipid oxidation are managed by metabolizing mechanisms. Since the in vivo signal is determined by the balance between production and elimination of the light-emitting molecules, cautions must be taken in the quantification of SCL as an indicator of oxidative stress, in particular concerning its kinetic features. For comparison of different samples, it seems necessary to perform the measurements at the same time after the oxidative treatment or alternatively to characterize the time-dependence of the luminescence emission. It is clear that the identification of the compounds responsible for this lipid peroxidation-associated chemiluminescence as well as the regulation of their concentration in vivo will be a major challenge in future studies of this signal in plants. 

## Data Availability

The data is contained within the article.
